# Use of Non-invasive Parameters and Machine-Learning Algorithms for Predicting Future Risk of Type 2 Diabetes: A Retrospective Cohort Study of Health Data From Kuwait

**DOI:** 10.3389/fendo.2019.00624

**Published:** 2019-09-11

**Authors:** Bassam Farran, Rihab AlWotayan, Hessa Alkandari, Dalia Al-Abdulrazzaq, Arshad Channanath, Thangavel Alphonse Thanaraj

**Affiliations:** ^1^Research Division, Dasman Diabetes Institute, Kuwait City, Kuwait; ^2^Department of Primary Health Care, Ministry of Health, Kuwait City, Kuwait; ^3^Department of Pediatrics, Farwaniya Hospital, Al Farwaniyah, Kuwait; ^4^Department of Pediatrics, Faculty of Medicine, Kuwait University, Kuwait City, Kuwait

**Keywords:** body mass index, prognosis, type 2 diabetes, hypertension, logistic regression, support vector machine, k-nearest neighbours

## Abstract

**Objective:** In recent decades, the Arab population has experienced an increase in the prevalence of type 2 diabetes (T2DM), particularly within the Gulf Cooperation Council. In this context, early intervention programmes rely on an ability to identify individuals at risk of T2DM. We aimed to build prognostic models for the risk of T2DM in the Arab population using machine-learning algorithms vs. conventional logistic regression (LR) and simple non-invasive clinical markers over three different time scales (3, 5, and 7 years from the baseline).

**Design:** This retrospective cohort study used three models based on LR, *k*-nearest neighbours (k-NN), and support vector machines (SVM) with five-fold cross-validation. The models included the following baseline non-invasive parameters: age, sex, body mass index (BMI), pre-existing hypertension, family history of hypertension, and T2DM.

**Setting:** This study was based on data from the Kuwait Health Network (KHN), which integrated primary health and hospital laboratory data into a single system.

**Participants:** The study included 1,837 native Kuwaiti Arab individuals (equal proportion of men and women) with mean age as 59.5 ± 11.4 years. Among them, 647 developed T2DM within 7 years of the baseline non-invasive measurements.

**Analytical methods:** The discriminatory power of each model for classifying people at risk of T2DM within 3, 5, or 7 years and the area under the receiver operating characteristic curve (AUC) were determined.

**Outcome measures:** Onset of T2DM at 3, 5, and 7 years.

**Results:** The k-NN machine-learning technique, which yielded AUC values of 0.83, 0.82, and 0.79 for 3-, 5-, and 7-year prediction horizons, respectively, outperformed the most commonly used LR method and other previously reported methods. Comparable results were achieved using the SVM and LR models with corresponding AUC values of (SVM: 0.73, LR: 0.74), (SVM: 0.68, LR: 0.72), and (SVM: 0.71, LR: 0.70) for 3-, 5-, and 7-year prediction horizons, respectively. For all models, the discriminatory power decreased as the prediction horizon increased from 3 to 7 years.

**Conclusions:** Machine-learning techniques represent a useful addition to the commonly reported LR technique. Our prognostic models for the future risk of T2DM could be used to plan and implement early prevention programmes for at risk groups in the Arab population.

## Introduction

During the last two decades, the Arab world, and particularly countries in the Gulf Cooperation Council, has experienced an unprecedented increase in the prevalence of type 2 diabetes mellitus (T2DM). Wealth accumulated during the oil era in these countries contributed to rapid urbanisation resulting in dramatic changes in dietary habits and lifestyle. The resultant population-wide increase in sedentary lifestyle habits was a major contributor to the high prevalence of T2DM (1, 2).

Early intervention and prevention strategies are needed to curb this urgent health crisis, and such strategies rely on the ability to identify individuals at future risk of diabetes. Previous reported trials, including studies of impaired glucose tolerance (IGT) testing and fasting biomarker levels (3), have demonstrated that lifestyle modifications or the use of medication can substantially reduce the risk of T2DM in people with IGT or elevated fasting and post-load plasma glucose concentrations (4, 5). However, these tests are relatively invasive, time-consuming, costly and inconvenient. Therefore, diabetes risk models based on known non-invasive risk factors and statistical analyses have been generated to identify individuals at future risk of developing T2DM (6–8). Such prognosis models can help to correctly identify individuals who should be targeted by intervention programmes and to avoid burdening low-risk individuals with invasive assessments, prevention, and treatment regimens. In other words, such models could improve the efficacy and cost-effectiveness of T2DM prevention programmes.

Existing diabetes prognosis models differ in terms of the extent of prediction horizons, techniques and types of assessed variables. Some models are based on basic non-invasive parameters, while others include invasive biomarkers. Although, the former may be more successful (7), the latter are more easily implementable and convenient on a large scale. Furthermore, previous studies have demonstrated good discrimination when using non-invasive models to predict the future risk of incident T2DM over a 10-years period; for example, Abbasi et al. (7) reported that the most basic prediction models (i.e., those that use non-invasive parameters) could distinguish people at high risk of developing diabetes within a time span of 5–10 years but not in shorter timeframes. However, most basic models overestimate the actual risk of diabetes. Abbasi et al. concluded that although existing prediction models could successfully distinguish individuals at high risk, they could not adequately quantify the actual future risk of diabetes.

Existing models are also limited by the fact that most were generated using populations of white American or European populations, and only a few have been externally validated in different populations (6). A risk score tends to exhibit a weaker discriminatory performance in an external population while overestimating the risk in the initial target population (9). Therefore, model performance must be evaluated broadly within the intended target population. In the Arab world, the prevalence of obesity is very high, and diabetes is more frequently associated with obesity (10) than with β-cell dysfunction (11). Obesity contributes significantly to T2DM pathogenesis through various mechanisms. In addition to the risk of diabetes, obesity increases the risks of developing hypertension, cardiovascular disease and some types of cancers, which account for ~50% of all deaths in the Arab region (12). Obesity-linked diabetes is a preventable disease, and a reduction in body weight decreases the risk of T2DM and its complications (13–15). These findings have encouraged collaboration among decision-makers from different Arab nations to limit the rise in obesity-related diabetes (10).

A number of mathematical techniques are used by researchers to build prognostic and predictive models in the field of biomedical applications. Apart from the techniques of logistic and Cox regression models that are often used in the field (7), machine-learning techniques have been demonstrated to have great potential (16, 17). Such machine-learning techniques include random forest (18), boosted regression tree (19), k-nearest neighbour (20), and support vector machines (SVMs) (21). Of these, the k-nearest neighbour and support vector machine are simplest classification systems having good discriminatory power (9).

In this study, we used data from a native Kuwaiti Arab population to develop prognostic models that could predict the risk of developing diabetes within three different time frames (3, 5, and 7 years) according to the body mass index (BMI) measurement at a given age. We implemented SVMs (22), k-nearest neighbours (k-NN) (23), and logistic regression (LR) (24) techniques to develop three models based on non-invasive parameters, which are age, BMI, family history of diabetes and hypertension, sex, and pre-existing hypertension.

## Materials and Methods

### Data From the Kuwait Health Network

The data used in this study were extracted from the Kuwait Health Network (KHN), which was collaboratively developed by the Dasman Diabetes Institute, Ministry of Health and the Public Authority of Civil Information of Kuwait. This network integrated health data from primary health centres and hospitals across Kuwait (9). The state clinics provide free primary health care and are located in all residential areas throughout Kuwait. Services include medical and dental care. These clinics are equipped to handle emergencies, as well as routine medical problems. The clinic has its own doctor or general practitioner who either provides treatment at the clinic or refer to one of the general hospitals. The primary contact for the patients and diagnosis are carried out at the primary health care centres; KHN integrates the patient data from these centres with data from hospital information system and laboratory information system when available for the patients. Thus, all the participants are from primary health centres.

The data records forming the research extract of the KHN were retrospective over a 9-year period, and all patients' names and civil identification numbers were anonymised before the data were provided to the researchers. Access to data from the KHN was approved by the Ethical Review Committee at the Dasman Diabetes Institute.

### Data Content

The research extract from the KHN contains data on 107,821 native Kuwaiti participants without T2DM and 40,773 native Kuwaiti patients with T2DM. The participants without diabetes visit primary health centres and hospitals for various other ailments. The diagnosis for diabetes is generally carried out at the primary health centres (and then were referred to hospitals, if required) unless the participants were already visiting the hospitals for other ailments. The diagnoses of diabetes and hypertension were ascertained through clinical procedures. The outcome, T2DM, was defined from clinical records. The diagnosis was validated by way of using recorded blood glucose levels during visits at and around the diagnosis of diabetes. The extracted data included demographic information, anthropometric values, vital signs, and clinical laboratory measurements (the latter values were sparse). Not all data items were available for all the participants. This limited the size of the data pool and restricted the number of study subjects. It is possible that patients with missing data have different risk profiles as compared with patients included; however, the missing data were most often due to the reason that the integration of data by KHN was partial and ongoing.

The present study applied an inclusion criterion of a visit by the participant to a primary care centre and/or hospital at least 3 years prior to the diagnosis of T2DM with a recorded BMI measurement; as regards the participants marked as controls, it was required that the participant was continuously monitored for diagnosis of diabetes over at least 7 years since the first visit with recorded BMI measurement. This has markedly reduced the number of study subjects eligible for the study. The resultant data set comprised 1,837 native Kuwaiti patients with complete records of the following measurements: sex, ethnicity, family history of hypertension, family history of diabetes, pre-existing hypertension, BMI measurement (kg/m^2^) and date of measurement (i.e., study entry point), age at BMI measurement and interval between the date of BMI measurement and diagnosis of diabetes (in months). Family histories of diabetes and hypertension were limited to first-degree relatives. Pre-existing hypertension was ascertained from clinical diagnostic data. The time point of obtaining the data for predictor variables such as sex, ethnicity, family history, and pre-existing condition of hypertension were at the study entry point (date of measurement of the first BMI measurement).

### Classification of Data Sets for the Study

For categorizing participants according to BMI, the classification system approved by the WHO was used: normal weight (BMI = 18.5–24.9 kg/m^2^), overweight (25.0–29.9 kg/m^2^), mildly obese (30.0–34.9 kg/m^2^), moderately obese (35.0–39.9 kg/m^2^), and severely obese (≥40 kg/m^2^). For categorizing participants according to age, the following classification system commonly used in the community [example as in: “Middle Age: definition of middle age in Oxford dictionary (American English) (US)”. *Oxforddictionaries.com*. Retrieved 2018-11-09] was adopted: adolescence (13–19 years), early adulthood (20–45 years), middle adulthood (45–65 years), and old age (>65 years).

### Statistical Analysis

Data mining and machine-learning calculations were performed using software from the R Project for Statistical Computing (https://www.r-project.org/). The models used in this study were explained in our previous publication (9) and are summarised below:

#### Logistic Regression

LR describes the relationship between an event and one or more independent variables by estimating probabilities and is used to formulate a generalised linear model. The number of regression coefficients corresponds to the number of measurements related to each hospital visitor. This statistical technique is widely used in the field of health research (24) to explain the associations among a set of explanatory variables with a binary response variable. The association of predictors with the diabetes status is measured using Odds Ratio (OR).

#### k-Nearest Neighbours

The k-NN is a simple classification algorithm that searches an entire training set for the *k*-closest neighbours and classifies new cases based on a majority vote (23). To determine closeness, Euclidean distance is used in the case of continuous variables and Hamming distance for binary data. We used the caret package in R and five-fold cross-validation to test multiple values of *k* and determine the optimal value for the data.

#### Support Vector Machine

SVM is a supervised machine-learning technique based on supervised learning algorithms and used for classification and regression analyses. The classification algorithm learns from the data input (e.g., health records of patients with and without diabetes) and divides the data into two categories (e.g., diabetic and non-diabetic groups) by maximising the margin between the support points. Subsequently, the algorithm predicts which of the two possible classes should include each new data point. The success of SVM can be attributed to its ability to maximise the *margin*, which denotes the distance between an example and the decision boundary (22). As the unseen examples (test cases) will be similar to the training examples, this large margin ensures better generalisation to the test cases. The programme is set to select arbitrary values for a cost variable, C, which controls the trade-off between training errors and margin maximisation.

#### Denoting Discriminatory Power of the Models

The performance of a model was assessed by way of calculating the area under the receiver operating characteristic (ROC) curve (AUC). ROC curves compare sensitivity vs. specificity across a range of values for the ability to predict a dichotomous outcome. It is one the most common statistical technique used to quantify how well the model can distinguish between two states, i.e., in the context of the presented study, people who will or will not develop diabetes in the prediction horizon.

## Results

### Descriptive Statistics of the Data Sets Used for the Analysis

The descriptive statistics of the participants are presented in Table 1. The cohort comprised 1,837 native Kuwaiti Arab participants (of whom 49.5% were men) with a complete record of the following measurements: age at baseline, BMI, family history of diabetes, family history of hypertension, diagnosis of hypertension, sex, and the time interval (in months) from the time of study entry to diabetes diagnosis (the last variable was not included in the analysis).

**Table 1:** Descriptive statistics of the participants.

Total number of participants	1,837
Sex (Male:Female)	909:928 (49.5%:50.5%)
Number of participants with a family history of T2DM	587 (32.0%)
Number of participants with a family history of hypertension	371 (20.2%)
Number of participants who were baseline hypertensive	1,316 (71.6%)
Number of participants with T2DM considering the 3-year horizon	290 (15.8%)
Number of participants with T2DM considering the 5-year horizon	468 (25.5%)
Number of participants with T2DM considering the 7-year horizon	647 (35.2%)
Mean age of participants at T2DM onset considering the 3-year horizon (years)	55.1 ± 11.0
Mean age of participants at T2DM onset considering the 5-year horizon (years)	56.7 ± 11.5
Mean age of participants at T2DM onset considering the 7-year horizon (years)	58.4 ± 11.5
Mean BMI of participants considering the 3-year horizon (kg/m^2^)	33.6 ± 10.2
Mean BMI of participants considering the 5-year (kg/m^2^)	33.2 ± 8.9
Mean BMI of participants considering the 7-year horizon (kg/m^2^)	33.0 ± 8.5
Mean interval from study entry point to diabetes diagnosis considering the 3-year horizon (months)	17.9 ± 11.5
Mean interval from study entry point to diabetes diagnosis considering the 5-year horizon (months)	29.6 ± 17.9
Mean interval from study entry point to diabetes diagnosis considering the 7-year horizon (months)	41.5 ± 24.8

*T2DM, type 2 diabetes mellitus; BMI, body mass index*.

Of these 1,837 participants, 647 had developed diabetes within 7 years since the date of the baseline BMI measurement (including 290 and 468 who had developed diabetes within 3 and 5 years, respectively). The remaining 1,190 participants did not develop diabetes even after 7 years since the date of the baseline measurement.

At baseline, the mean age of all participants was 59.5 ± 11.4 years, The participants exhibited the following distribution into age categories at baseline: adolescence: 2 (0.1%); early adulthood: 144 (7.8%); middle adulthood: 1,116 (60.8%); and old age: 575 (31.3%). Patients who developed T2DM were more often from middle adulthood age group: adolescence: 2 (0.3%); early adulthood: 90 (13.9%); middle adulthood: 446 (68.9%); and old age: 109 (16.8%).

At baseline, the participants had a mean BMI of 31.6 ± 7.1 kg/m^2^, and were distributed into the following BMI categories: underweight: 0.3%; normal weight: 12.1%; overweight: 34.1%; mildly obese: 28.3%; and moderately and severely obese: 25.2%. In the overall data set, 587 and 371 of the participants had a family history of diabetes or hypertension, respectively, and 71.6% (1316/1837) were hypertensive at baseline. For comparison, 41% of the 40,773 T2DM patients present in the initial research extract from KHN presented with comorbid hypertension. Higher proportion of the study individuals being hypertensive is because the study participants are predominantly from late adulthood and old age–hypertension is typically prevalent in such an age group.

Table 2 presents comparative descriptive statistics between the group of participants with T2DM onset within 7 years since the study entry point and the group of participants without onset of diabetes within the same time duration. The mean age and BMI at study entry point, pre-existing condition of hypertension and family history of hypertension were significantly different between the two groups. The mean age at the baseline BMI measurement was 54.9 ± 11.1 years among those who developed T2DM and 61.9 ± 10.8 years among those who did not develop T2DM. Notably, 67% of participants that developed T2DM within 7 years and 74% of those that did not develop T2DM were hypertensive at baseline. The mean BMI at study entry point was significantly higher for individuals who developed T2DM within 7 years compared to those who did not develop T2DM (32.95 ± 8.45 vs. 30.82 ± 6.19; *p* < 0.001).

**Table 2:** Descriptive statistics of participants who became diabetic within 7 years since study entry point vs. those who did not become diabetic.

	**Diabetic group (*n* = 647)**	**Non-diabetic group (*n* = 1,190)**	***p*-value**
Male	311 (48.1%)	598 (50.3%)	0.4
Mean age at entry point (years)	54.92 ± 11.05	61.9 ± 10.8	<0.001
Mean BMI at entry point (kg/m^2^)	32.95 ± 8.45	30.82 ± 6.19	<0.001
Positive diagnosis for hypertension at entry point	431 (66.6%)	885 (74.3%)	<0.001
Family history of diabetes	191 (29.5%)	396 (33.2%)	0.110
Family history of hypertension	165 (25.5%)	206 (17.3%)	<0.001

### Prognostic Models for T2DM

We constructed three different models using the LR, k-NN, and SVM techniques for predictions over three different time horizons: 3, 5, and 7 years.

#### 3-Year Prediction Horizon

**Figure 1:**
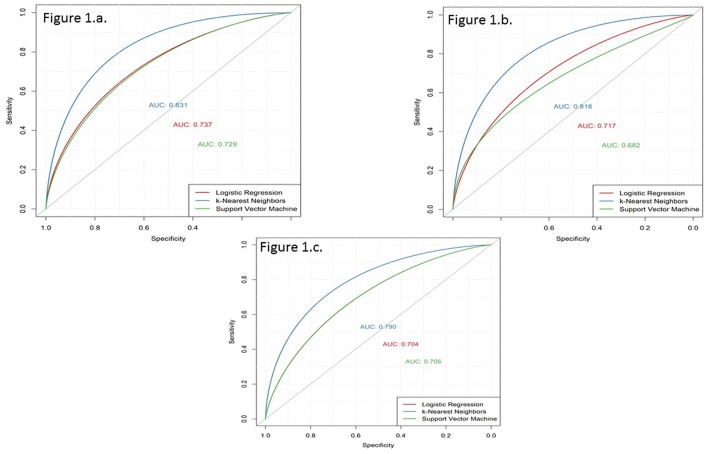
Receiver operating characteristic (ROC) curves derived for prediction horizons of 3, 5, and 7 years using the three models based on logistic regression (LR), k-nearest neighbours (k-NN), and support vector machine (SVM). **(a)** 3-year prediction horizon. **(b)** 5-year prediction horizon. **(c)** 7-year prediction horizon.

Two hundred and ninety cases and 1,547 controls were available for the 3-year prediction horizon. The performance results of the three models are presented in Table 3 and Figure 1A. Using the LR function and five-fold cross-validation, we achieved an AUC value of 0.737. Using the SVM technique (with default parameters), we achieved a similar AUC value (0.729). The use of five-fold cross-validation to select the optimal hyper-parameter for k-NN yielded a k value of 8 and an AUC of 0.831, which was significantly better than the LR and SVM models. In other words, the k-NN model was significantly discriminatory.

**Table 3:** AUC values obtained using logistic regression, k-nearest neighbours, and Support vector machine models designed for predicting the risk of T2DM over three different prediction horizons.

**Prediction horizons**	**Logistic regression**	**k-nearest neighbours**	**Support vector machine**
3-year	0.737 95% CI: 0.7049–0.7692	0.8308 95% CI: 0.8079–0.8537	7286 95% CI: 0.696–0.7611
5-year	0.7161 95% CI: 0.6886–0.7435	0.818 95% CI: 0.7973–0.8389	0.6823 95% CI: 0.6514–0.7132
7-year	0.7039 95% CI: 0.6791–0.7286	0.7903 95% CI: 0.7694–0.8112	0.7059 95% CI: 0.6812–0.7306

*AUC, area under the curve; T2DM, type 2 diabetes mellitus; CI, confidence interval*.

The 1,547 individuals forming the controls are those that did not develop T2DM in the 3-years period from study entry point; the results did not differ when we experimented with having only the 1,190 individuals, who did not develop T2DM during the entire study period, as controls.

#### 5-Year Prediction Horizon

Four hundred and sixty eight cases and 1,369 controls were available for the 5-year prediction horizon. The performance results of the three models are presented in Table 3 and Figure 1B. The discrimination of the LR model did not change when applied to a 5-year prediction horizon, which yielded a slightly lower AUC (0.716) than what was obtained for the 3-year prediction horizon. Similarly, although the discriminatory power of the SVM model decreased slightly from 3 to 5 years (0.729–0.682, respectively), this decrease was not statistically significant (*p* = 0.08). By contrast, a five-fold cross-validation yielded a k value of 8 and an AUC of 0.818, which was slightly lower than that obtained with the 3-year prediction horizon. Again, the k-NN method performed better than the LR and SVM models.

The 1,369 individuals forming the controls are those that did not develop T2DM in the 5-years period from study entry point; the results did not differ when we experimented with having only the 1,190 individuals, who did not develop T2DM during the entire study period, as controls.

#### 7-Year Prediction Horizon

Six hundred and forty-seven cases and 1,190 controls were available for the 7-year prediction horizon. The performance results of the three models are presented in Table 3 and Figure 1C. Here, the LR model yielded a lower AUC (0.70) for the 7-year prediction horizon than for the 3- (0.74) and 5-year horizons (0.72). The SVM model yielded an AUC of 0.71 at 7 years, which was slightly higher and lower than the values obtained for the 5- and 3-year horizons, respectively (0.68 and 0.73, respectively). Although, a five-fold cross-validation for k-NN hyper-parameter selection again yielded a k value of 8, this method yielded a lower discriminatory power (AUC = 0.79) for the 7-year horizon relative to the 5- (0.82) and 3-year (0.83) horizons. Still, the k-NN model performed better than the LR and SVM models.

### Coefficients Identified as Significant When Applying Logistic Regression Model to the Three Prediction Horizons

Among the multiple LR coefficients deduced with the models for 3- and 5-year prediction horizons, sex, and family history of diabetes were insignificant factors (Table 4); however the family history of diabetes became a significant variable in the 7-year model, and sex remained the only insignificant variable for this time horizon.

**Table 4:** Variables identified as significant (shown in bold) when applying the logistic regression model to the three prediction horizons.

**Terms from logistic regression model**	**Coefficient**	**Odds ratio (95% CI)**	***p*-value**
**3-YEAR PREDICTION HORIZON**			
**Constant**	**1.636**		**0.007**
**Baseline age**	**−0.068**	**0.93 (0.92, 0.94)**	**<0.001**
**Baseline BMI**	**0.0357**	**1.06 (1.04, 1.08)**	**<0.001**
Family history of diabetes	−0.094	0.89 (0.67, 1.17)	0.547
**Family history of hypertension**	**0.984**	**1.73 (1.28, 2.33)**	**<0.001**
Diagnosis for baseline hypertension	**−0.557**	**0.57 (0.44, 0.75)**	**<0.001**
Sex	−0.11	0.99 (0.77, 1.28)	0.447
**5-YEAR PREDICTION HORIZON**			
**Constant**	**1.937**		**<0.001**
**Baseline age**	**−0.0623**	**0.94 (0.93, 0.95)**	**<0.001**
**Baseline BMI**	**0.031**	**1.05 (1.03, 1.07)**	**<0.001**
Family history for diabetes	−0.1523	0.89 (0.71, 1.12)	0.239
**Family history of hypertension**	**0.853**	**1.61 (1.25, 2.08)**	**<0.001**
Diagnosis for baseline hypertension	**−0.408**	**0.63 (0.5, 0.8)**	**<0.01**
Sex	−0.171	0.92 (0.75, 1.15)	0.156
**7-YEAR PREDICTION HORIZON**			
**Constant**	**2.0934**		**0.0000**
**Baseline age**	**−0.0589**	**0.94 (0.93, 0.95)**	**0.0000**
**Baseline BMI**	**0.0295**	**1.05 (1.03, 1.06)**	**0.0004**
**Family history of diabetes**	**−0.2389**	**0.84 (0.68, 1.03)**	**0.0400**
**Family history of hypertension**	**0.8325**	**1.64 (1.3, 2.06)**	**0.0000**
Diagnosis for baseline hypertension	**−0.3106**	**0.69 (0.56, 0.85)**	**0.0137**
Sex	−0.1650	0.92 (0.76, 1.11)	0.1261

*BMI, body mass index*.

### Other Performance Metrices (Such as Sensitivity and Specificity) for the Models

In order to derive other performance metrices such as sensitivity and specificity of the model, we matched the sizes of the case and control. Best measure for sensitivity was obtained with SVM model at 44% for 7-year prediction horizon and at 35% for 5-year prediction horizon (Table S1). AUC was always higher with k-NN model in all the three prediction horizons, but the accuracy was comparatively lower for k-NN. This is because the measures of sensitivity, specificity and accuracy characterize the true positive rate and true negative rate at the threshold value of 0.5, while AUC is computed by adding all the “accuracies” computed for all the possible threshold values. Hence, AUC is an average (expected value) of those accuracies when computed for all threshold values.

## Discussion

As noted, Arab countries have seen increase in the incidence rates of obesity, diabetes, and metabolic syndrome (25–28) in recent decades. Accordingly, tools that can be used to accurately identify high-risk individuals as targets for early intervention and prevention programmes are urgently needed. In this retrospective cohort study, we analysed a data set from native Arab individuals who were predominantly middle-aged and older and among whom more than half were either obese or very obese. We demonstrated that prognostic models developed using six non-intrusive parameters (baseline age, BMI, family histories of diabetes and hypertension, pre-existing hypertension, and sex) could identify patients at a high risk of developing T2DM within 3–7 years. In our study, the k-NN machine-learning technique outperformed the most commonly used LR, as well as another tested model based on SVM. For all models, however, the discriminatory power decreased as the prediction horizon increased.

Table 5 lists the previously published T2DM risk assessment tools and classification models developed using non-invasive parameters. These studies, which were mostly based on LR models, yielded AUC discrimination values of 0.76–0.78, which were lower than the values obtained with our k-NN models (0.79–0.83) but higher than the values obtained with our LR (0.70–0.74) and SVM models (0.70–0.73). We note that all previously reported studies included lifestyle data (e.g., smoking, physical activity, diet, and medication) in addition to the standard non-invasive parameters used in our models. Accordingly, our newly developed k-NN model outperformed these reported studies.

**Table 5:** Comparison of the presented prognostic models with models reported in the literature.

	**Study title, citation (prediction horizons)**	**Model; outcome measure; population; sample size**	**Predictors used**	**AUC**
1	Current study (3, 5 and 7 years)	k-nearest neighbours; Reports AUC; Population: native Arabs from Kuwait; Sample size: 1,837	Age, BMI, family history of diabetes, hypertensive status, family history of hypertension, and sex	0.83 (3-year), 0.82 (5-year), 0.79 (7-year)
2	Current study (3, 5 and 7 years)	Logistic regression; Support vector machine; Reports AUC; Population: native Arabs from Kuwait; Sample size: 1,837	Age, BMI, family history of diabetes, hypertensive status, family history of hypertension, and sex	LR: 0.74 (3-year), 0.72 (5-year), 0.70 (7-year) SVM: 0.73 (3-year), 0.68 (5-year), 0.71 (7-year)
3	Alssema et al. (29): “The evaluation of screening and early detection strategies for type 2 diabetes and impaired glucose tolerance (DETECT-2) update of the Finnish diabetes risk score for prediction of incident type 2 diabetes” (5 years)	Logistic regression; Reports AUC; Population: Finnish; Sample size: 18,301	Age, BMI, Waist circumference, physical activity, diet, use of antihypertensive medication, history of high blood glucose level, sex, smoking, and family history of diabetes (parent, sibling, or both)	0.77
4	Wannamethee et al. (30): “The potential for a two-stage diabetes risk algorithm combining non-laboratory-based scores with subsequent routine non-fasting blood tests: results from prospective studies in older men and women” (7 years)	Logistic regression; Reports AUC; also reports sensitivity and specificity in the top quintile of the score; Population: British; Sample size: 3,523 men and 3,404 women	Age, sex, family history of diabetes, smoking status, BMI, waist circumference, and hypertension	AUC: 0.77. Sensitivity: 50.3%; specificity: 81.4%
5	Rathmann et al. (31): “Prediction models for incident type 2 diabetes mellitus in the older population: KORA S4/F4 cohort study” (6 years)	Logistic regression; Reports AUC; Population: Germans; Sample size: 1,353	Age, sex, BMI, parental diabetes, smoking, and hypertension	0.76
6	Chen et al. (32): “AUSDRISK: an Australian Type 2 Diabetes Risk Assessment Tool based on demographic, lifestyle and simple anthropometric measures” (5 years)	Logistic regression; Reports AUC; also sensitivity, specificity, and positive predictive values; Population: more than 85% of participants were born in Australia, New Zealand, or the United Kingdom; Sample size: 6,060	Age, sex, ethnicity, parental history of diabetes, history of high blood glucose level, use of antihypertensive medications, smoking, physical inactivity, and waist circumference	AUC = 0.78. Sensitivity = 74% Specificity = 68% Positive predictive value = 13%
7	Rosella et al. (33): “A population-based risk algorithm for the development of diabetes: development and validation of the Diabetes Population Risk Tool (DPoRT)” (9 years)	Logistic regression; Reports C-statistics which is AUC; Population: residents of Ontario in Canada; Sample size: 19,861	BMI, age, ethnicity, hypertension, immigrant status, smoking, education status, and heart disease	0.77
8	Joseph et al. (34): “Incidence of and risk factors for type-2 diabetes in a general population: The Tromsø study” (6 years)	Cox proportional hazard models; Reports hazards ratio; Population: Caucasian subjects from Norway; Sample size: 12,431 men and 13,737 women	Age, BMI, triglycerides, high-density lipoprotein cholesterol, hypertension, family history of diabetes, low education, and smoking	–
9	Kahn et al. (35): “Two risk-scoring systems for predicting incident diabetes mellitus in U.S. adults age 45 to 64 years” (10 years)	Proportional hazard models; Reports risk score derived from proportional hazard coefficients; Population: USA adults with European or African ancestry; Sample size: 12,729	Waist circumference, maternal diabetes, hypertension, paternal diabetes, short stature, black race, age 55 years or older, increased weight, rapid pulse, and smoking history	–
10	Hippisley-Cox et al. (36): “Predicting risk of type 2 diabetes in England and Wales: prospective derivation and validation of QDScore” (10 year)	Cox proportional hazards models; Reports hazard ratios; Population: multi-ethnic from UK; Sample size: 2,540,753 (Model development); 1,232,832 (Model validation)	Ethnicity, age, sex, body mass index, smoking status, family history of diabetes, townsend deprivation score, treated hypertension, cardiovascular disease, and current use of corticosteroids	–
11	Balkau et al. (37): “Predicting diabetes: clinical, biological, and genetic approaches: data from the Epidemiological Study on the Insulin Resistance Syndrome (DESIR)” (9 years)	Logistic regression; Reports AUC; Population: French; Sample size: 1,863 men and 1,954 women	Waist circumference and hypertension in both sexes, smoking in men and diabetes in the family in women	0.71 for men, 0.83 for women
12	Simmons et al. (38): “Do simple questions about diet and physical activity help to identify those at risk of Type 2 diabetes?” (5 years)	Logistic regression; Reports AUC; Population: British; Sample size: 25,633	Physical activity, diet, age, BMI, and family history	0.76
13	Wilson et al. (39): “Prediction of incident diabetes mellitus in middle-aged adults. The Framingham offspring study” (7 years)	Logistic regression; Reports AUC; Population: white and non-Hispanic; Sample size: 3,140	Age, sex, parental history of diabetes, and BMI	0.72
14	Lindström et al. (40): “The diabetes risk score: a practical tool to predict type 2 diabetes risk” (10 years)	Logistic regression; Reports AUC and sensitivity and specificity; Population: Finnish; Sample size: 4,746 (Model development); 4,615 (Model validation)	Age, BMI, waist circumference, history of antihypertensive drug treatment, high blood glucose, physical activity, and diet	0.85 (model development); (0.87) model validation. Sensitivity, 0.78; specificity, 0.77

The uses of tools such as the presented models are two-fold. First, at the individiuals level, these models identify subjects at high risk for T2DM; such subjects can be targeted for prevention programmes that address issues such as awareness, fitness and nutrition. At the population level, the application of these programmes would greatly reduce the national economic burden associated with diabetes care, as these programmes are far less expensive than the treatment of diabetes and its complications. Second, patients identified as high-risk for developing T2DM comprise an interesting cohort from a research perspective. These patients can be monitored in the context of prevention programmes, using more detailed data (such as biochemical markers). Furthermore, these risk assessment tools can also be introduced to the public via online platforms, which would allow individuals to check their risk levels from the comfort of their homes. Such platforms could decrease the number of low-risk patients visiting healthcare facilities and increase the number of high-risk patients that might otherwise have remained ignorant of their risk status. These latter patients could then be invited to participate in a more detailed assessment based on invasive biomarkers.

Interestingly, pre-existing condition of hypertension was associated with a reduction in the likelihood of developing diabetes in all the three prediction horizons (see Table 4) though it is known that hypertension and diabetes are “concordant” disorders and represent parts of the overall identical pathophysiological risk profile (41); Positive family history of hypertension was associated with higher odds of developing diabetes in all the three prediction horizons (Table 4). Positive family history of diabetes was associated with lower odds of developing diabetes in all the three prediction horizons, though the odds ratio was statistically significant only in the case of 7-year prediction horizon. In a similar manner, increased age at baseline was associated with a lesser risk of developing diabetes (Table 4); the study subjects were mostly from the higher risk group of late adulthood and old age (mean age of all participants at baseline was 59.5 ± 11.4 years) and the mean age at baseline was significantly higher for individuals who did not develop diabetes compared to that for individuals who developed diabetes within 7 years from study entry point (61.9 ± 10.8 vs. 54.92 ± 11.05; *p* < 0.001) (Table 2). The observed inverse association with age can be partly explained by the general observations (from literature) that in Kuwait the age of onset of T2DM is relatively low—for example, we have earlier reported the mean onset age for T2DM from a larger data set of Kuwaiti natives as 48.63 ± 12.12 years (25).

As mentioned earlier, ROC is the standard technique used to measure test performance and to compare the performance among different models. However, to understand the clinical applicability of the developed models, reporting other performance metrics such as sensitivity and specificity is becoming necessary (42). Our work identified that the best value for sensitivity was obtained with SVM compared to the other two models (LR and k-NN) is only 44% (Table S1) and that if the prediction model is applied in clinical practice, it would mean than >50% of the cases will be missed. Though, the models are not readily usable for the clinical predictions, it is to be noted that the work illustrated that machine learning algorithms classify the subjects better than the logistic regression model. It is to be further noted that the models used only a small number of predictor variables (age, sex, BMI, family history of diabetes, pre-existing condition of hypertension, and family history of hypertension); making use of data (when available) on further predictor variables, such as on physical activity and lifestyle, in building the models is expected to improve the performance metrics.

Our study featured a notable strength. While most reported diabetes risk assessment tools were based on LR [see Table 5 and Abbasi et al. (7)], our study reported models based on machine-learning approaches, specifically the k-NN, with a consistently high discriminatory power. However, our study also had some limitations. First, the selection criteria of subjects for the study may cause bias in the dataset: (i) at baseline, the mean age of all participants was 59.5 ± 11.4 years, indicating that the study subjects are mostly from the higher risk group of late adulthood and old age who tend to seek medical care for pre-diabetes ailments. (ii) The subjects included in the study were required to have their BMI measurements recorded; physicians usually tend to check the BMI when they suspect obesity or other related ailments in the subjects; and hence the study subjects were already obese or over-weight leading to under-representation of normal-weight and lean subjects. Second, as up to 72% of the participants in this study had pre-existing hypertension at baseline, the study was limited by the absence of information regarding antihypertensive medication use; the observed higher prevalence of hypertension in the study subjects is not surprising as a large number of them were of late adulthood or old age and hypertension is prevalent in such an age group. Third, we did not have access to data regarding lifestyle factors, medication use, and other obesity indicators. Fourth, the models in this study did not account for variations in BMI that might occur during the interval from the date of the baseline measurement to the date of T2DM diagnosis, other changes (e.g., transition from non-hypertension to hypertension) or the administration of a new medication regimen or implementation of lifestyle modifications during the interim period. Fifth, extensive data quality assurance was missing. Finally, the predictive power of the models was not tested in the younger age group and was not validated in an external independent cohort.

In conclusion, our study findings demonstrate that the future risk of diabetes in Arab population can be predicted using non-invasive clinical parameters. Notably, our model based on the machine-learning technique k-NN outperformed those based on LR and SVM, as well as previously reported models, thus demonstrating the need to extend existing models using these machine-learning techniques. However, future studies should concentrate on developing similar models predicting future risk of T2DM in younger age groups to plan prevention programmes as early as possible.

## Author Contributions

TT undertook the study design, directed the reported work, and directed the development of the manuscript. BF performed all machine-learning algorithms and calculations. AC handled data extraction, created the different data sets, and performed the calculations. TT, BF, and AC developed the manuscript. RA represents Ministry of Health which participated in the generation of Kuwait Health Network data. RA, HA, and DA-A critically reviewed the manuscript and participated in the discussions.

### Conflict of Interest Statement

The authors declare that the research was conducted in the absence of any commercial or financial relationships that could be construed as a potential conflict of interest.

## Abbreviations

AUC: receiver operating characteristic curve
ROC: receiver operating characteristic
T2DM: type 2 diabetes mellitus
BMI: body mass index
k-NN: k-nearest neighbours
LR: logistic regression
SVM: support vector machine
KHN: Kuwait Health Network.
