# Supplementary material for: Use of Non-invasive Parameters and Machine-Learning Algorithms for Predicting Future Risk of Type 2 Diabetes: A Retrospective Cohort Study of Health Data From Kuwait
Source: Front Endocrinol (Lausanne). 2019 Sep 11;10:624. doi: 10.3389/fendo.2019.00624 (PMC6749017; doi:10.3389/fendo.2019.00624)
Supplement: Supplementary file 1 [file Table_1.DOCX]

**Supplementary Table S1: Performance metrics of the models for the three different prediction horizons.**

| **Metrices** | **Models (LR: logistic regression; k-NN: k-nearest neighbor; SVM: support vector machine)** | | |
| --- | --- | --- | --- |
|  | **LR** | **k-NN** | **SVM** |
| **3-years prediction horizon (290 cases, 297 controls)** | | | |
| Sensitivity | **33%** | 27% | **33%** |
| Specificity | 33% | 25% | 31% |
| AUC | 0.74 | **0.80** | 0. 74 |
| Accuracy | 33% | 26% | 32% |
| Brier Score | 0.67 | 0.74 | 0.68 |
| **5-years prediction horizon (468 cases, 476 controls)** | | | |
| Sensitivity | 33% | 27% | **35%** |
| Specificity | **34%** | 31% | 28% |
| AUC | 0.72 | **0.79** | 0.74 |
| Accuracy | 33.7% | 29% | 32% |
| Brier Score | 0.66 | 0.71 | 0.68 |
| **7-years prediction horizon (647 cases, 714 controls)** | | | |
| Sensitivity | 38% | 32% | **44%** |
| Specificity | **30%** | 25% | 27% |
| AUC | 0.71 | **0.79** | 0.71 |
| Accuracy | 34.1% | 28% | 35% |
| Brier Score | 0.66 | 0.72 | 0.65 |
